# Fabrication of Aluminum Tubes Filled with Aluminum Alloy Foam by Friction Welding

**DOI:** 10.3390/ma8105373

**Published:** 2015-10-23

**Authors:** Yoshihiko Hangai, Yukiko Nakano, Shinji Koyama, Osamu Kuwazuru, Soichiro Kitahara, Nobuhiro Yoshikawa

**Affiliations:** 1 Graduate School of Science and Technology, Gunma University, Kiryuu 376-8515, Japan; t14802062@gunma-u.ac.jp (Y.N.); koyama@gunma-u.ac.jp (S.K.); 2 Graduate School of Engineering, University of Fukui, Fukui 910-8507, Japan; kuwa@u-fukui.ac.jp; 3 Hokudai Co., Ltd., Abira 059-1434, Japan; soichiro_kitahara@hokudai-jp.com; 4 Institute of Industrial Science, University of Tokyo, Tokyo 153-8505, Japan; yoshi@telu.iis.u-tokyo.ac.jp

**Keywords:** cellular materials, composites, friction welding, X-ray computed tomography

## Abstract

Aluminum foam is usually used as the core of composite materials by combining it with dense materials, such as in Al foam core sandwich panels and Al-foam-filled tubes, owing to its low tensile and bending strengths. In this study, all-Al foam-filled tubes consisting of ADC12 Al-Si-Cu die-cast aluminum alloy foam and a dense A1050 commercially pure Al tube with metal bonding were fabricated by friction welding. First, it was found that the ADC12 precursor was firmly bonded throughout the inner wall of the A1050 tube without a gap between the precursor and the tube by friction welding. No deformation of the tube or foaming of the precursor was observed during the friction welding. Next, it was shown that by heat treatment of an ADC12-precursor-bonded A1050 tube, gases generated by the decomposition of the blowing agent expand the softened ADC12 to produce the ADC12 foam interior of the dense A1050 tube. A holding time during the foaming process of approximately *t*_H_ = 8.5 min with a holding temperature of 948 K was found to be suitable for obtaining a sound ADC12-foam-filled A1050 tube with sufficient foaming, almost uniform pore structures over the entire specimen, and no deformation and minimum reduction in the thickness of the tube.

## 1. Introduction

Aluminum foam is lighter than dense aluminum (Al) and exhibits superior energy absorption and sound insulation properties [1,2]; therefore, it is expected to be used in components of automobiles and construction materials. Al foam is usually used as the core of composite materials by combining it with dense materials, such as in Al foam core sandwich panels [3,4,5,6,7] and Al-foam-filled tubes [8,9,10,11,12,13,14,15], owing to its low tensile and bending strengths. These composite materials are usually fabricated by bonding an Al foam core to a dense material using an adhesive. However, the use of an adhesive prevents these composite materials from being used at high temperatures [4], decreases their recyclability [16], and has raised considerable environmental concerns [16]. Metal bonding between Al foam and a dense metal without using an adhesive has been realized by clad bonding [4,6] and friction stir welding (FSW) processes [17,18]. However, these processes are limited to the fabrication of flat sandwich panels, and little research on the fabrication of Al-foam-filled tubes with metal bonding has been reported.

Recently, a fabrication process for Al-foam-filled dense steel tubes with metal bonding has been developed [19,20]. In this process, using a friction-based process such as friction welding [21,22] or friction stir back extrusion [23,24,25,26,27], a solid Al composite in which blowing agent powder is uniformly distributed, called the precursor, is first bonded with a dense steel tube. In the heat treatment of the bonded precursor, gases generated by the decomposition of the blowing agent expand the softened Al to produce the Al foam interior of the dense steel tube. It was shown that an Fe-Al diffusion bonding layer with a thickness of approximately 10 μm was formed, indicating that metal bonding between the Al foam and steel tube was realized. In a previous study on Al foam core sandwich panels comprising one Al foam core layer and two dense SPCC low-carbon steel face sheet layers, although an Fe-Al intermetallic compound layer with a thickness of several tens μm was generated at the interface, the bonding of the interface exhibited higher strength than that of the Al foam itself [17]. However, an all-Al foam-filled tube instead of a steel tube is desirable to avoid the generation of a brittle intermetallic compound layer and to further reduce the weight and increase recyclability.

In this study, all-Al foam-filled tubes consisting of ADC12 Al-Si-Cu die-cast aluminum alloy (equivalent to A383.0 aluminum alloy) foam and a dense A1050 commercially pure Al tube with metal bonding were fabricated by friction welding. First, the deformation behavior of the ADC12 foam precursor during friction welding was nondestructively observed by X-ray computed tomography (CT). Then, the relationships between the deformation behavior of the precursor, the temperature, and the torque of the rotating tool during friction welding were investigated. Next, the holding time during the foaming process was investigated from the viewpoint of achieving sufficient foaming to obtain ADC12-foam-filled A1050 tubes with relatively good pore structures in the foam and no deformation or reduction in the thickness of the tube. The melting points are relatively similar for the interior and exterior of the tube (solidus and liquidus temperatures of ADC12 are 788 K and 853 K, respectively, and solidus and liquidus temperatures of A1050 are 933 K and 919 K, respectively) compared with the using case of a steel tube. It is therefore of concern that the A1050 tube may soften during the foaming process, causing its deformation and the infiltration of ADC12 foam into the tube. The pore structures and the reduction of the thickness of the A1050 tube of fabricated ADC12-foam-filled A1050 tubes were nondestructively observed by X-ray CT. In addition, the distribution of elemental Si, which only exists in ADC12 Al alloy at a concentration of approximately 10 mass %, was observed by electron probe microanalysis (EPMA).

## 2. Results and Discussion

### 2.1. Relationship between Deformation Behavior of ADC12 Precursor and Torque/Temperature during Friction Welding

**Figure 1:**
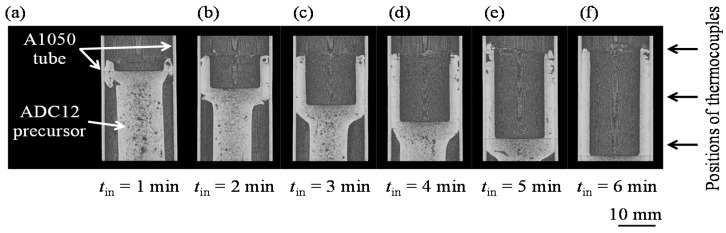
X-ray CT deformation images of the ADC12 precursor during friction welding with various tool indentation times *t*_in_. (**a**) *t*_in_ = 1 min; (**b**) *t*_in_ = 2 min; (**c**) *t*_in_ = 3 min (**d**) *t*_in_ = 4 min; (**e**) *t*_in_ = 5 min; (**f**) *t*_in_ = 6 min.

Figure 1 shows X-ray CT longitudinal-sectional deformation images of the ADC12 precursor in the A1050 tube during friction welding with various tool indentation times *t*_in_. The images were reconstructed from cross-sectional X-ray CT images of the entire specimen using ImageJ image processing software. *t*_in_ is the tool indentation time from when the tool first came into contact with the surface of the ADC12 precursor. Note that these images do not show the same sample, for example, for the sample with *t*_in_ = 2 min, a new ADC12 precursor and A1050 tube were prepared, then the tool indentation was conducted from *t*_in_ = 0 min to *t*_in_ = 2 min. When the ADC12 precursor was softened by the generated friction heat, the precursor deformed and came into contact with the A1050 tube in the vicinity of the rotating tool, as shown in Figure 1a. The contact area between the rotating tool and the deformed precursor and that between the deformed precursor and the A1050 tube increased as the indentation of the tool increased. The precursor mainly deformed in the vicinity of the rotating tool, and a gap remained between the precursor and the tube away from the tip of the rotating tool. Finally, as the tool approached the bottom of the tube, the gap between the precursor and the tube disappeared, as shown in Figure 1f. No deformation of the tube was observed at the resolution of X-ray CT used in this study.

**Figure 2:**
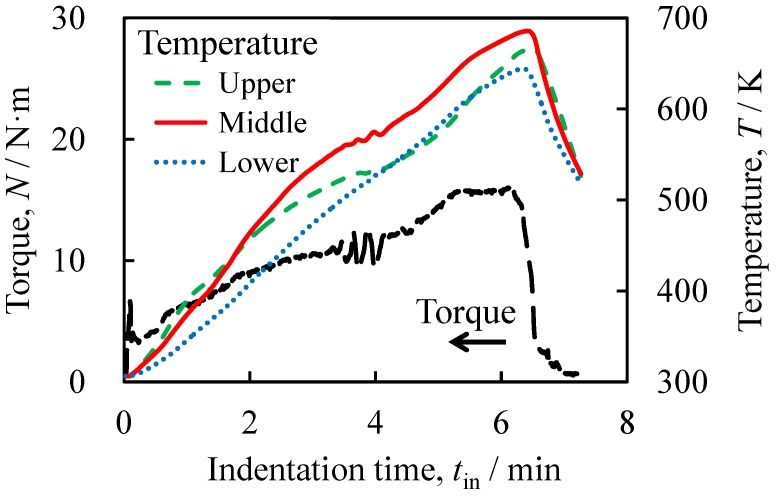
Torque of the rotating tool *N* and jig temperature *T* as functions of indentation time *t*_in_ during friction welding. The temperature was observed at upper, middle, and lower positions in the jig as shown in Figure 1.

Figure 2 shows the variations in the torque of the rotating tool *N* and the jig temperature *T* with respect to the indentation time *t*_in_ during the friction welding. The temperature was observed at upper, middle, and lower positions in the jig as shown in Figure 1. A shock torque appeared for a few seconds immediately after the contact between the tool and the ADC12 precursor. The increased contact area between the rotating tool and the deformed precursor as well as that between the deformed precursor and the A1050 tube with increasing indentation by the tool caused the increase in torque. Accordingly, the temperature increased with increasing indentation of the tool owing to the increased friction heat generated between the tool and the precursor as the contact area between them increased. In particular, the temperature at the upper and middle positions, which were in the vicinity of the rotating tool where the friction heat was generated, increased rapidly. The heat generated in the vicinity of the rotating tool, which was transferred to the A1050 tube and the fixing jig, gradually increased the temperature at the lower position. With increasing indentation of the rotating tool, on the one hand, the deformation resistance of the precursor increased owing to the increase in contact area; on the other hand, the deformation resistance of the precursor decreased owing to its increased temperature, which softened the precursor. The balance between the increase and decrease in deformation resistance caused the rate of increase of the torque to decrease. In contrast, the temperature continuously increased at all three positions with increasing indentation. As the tool indentation increased, the tip of the rotating tool where the friction heat was generated approached the lower position in the jig. Also, the friction between the side of the tool and the precursor further increased the amount of heat, resulting in an increase in the temperature at the middle and lower positions. In addition, the transfer of the generated heat through the precursor, the tube, and the fixing jig resulted in the temperature continuing to increase at the upper position. Although heat was released through the jig, the temperature at all three positions continuously increased during the indentation of the tool, because it is considered that the amount of heat released from the jig was less than that the amount of generated heat. When the tool reached the maximum indentation depth, the temperature reached maximum values of approximately 640–680 K at the three positions. Thereafter, the tool was moved upward and unloaded, causing the torque and temperature to decrease rapidly.

**Figure 3:**
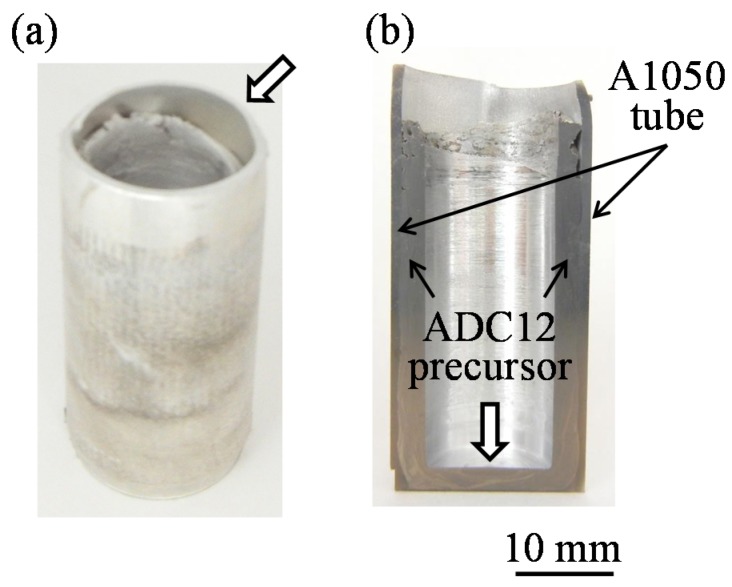
Fabricated ADC12-precursor-bonded A1050 tube. (**a**) Sample immediately after friction welding; (**b**) Cross section of sample in (**a**).

Figure 3a,b show a fabricated ADC12-precursor-bonded A1050 tube and its cross section, respectively. The bonded ADC12 precursor was found throughout the inner wall of the A1050 tube and no gap was observed, indicating that the precursor was attached firmly to the tube. In addition, no deformation of the tube was observed except at the top, where the precursor did not bond with the tube. Note that the top of the tube and the bottom of the precursor, indicated by wide arrows, were cut before the heating process.

It is considered that the temperatures at the points of contact between the rotating tool and the precursor were higher than those observed using the thermocouples, which were 2 mm away from the surface of the tube. However, as shown in Figure 3, foaming was not observed during friction welding.

### 2.2. Pore Structures of ADC12 Foam and Thickness of A1050 Tube

**Figure 4:**
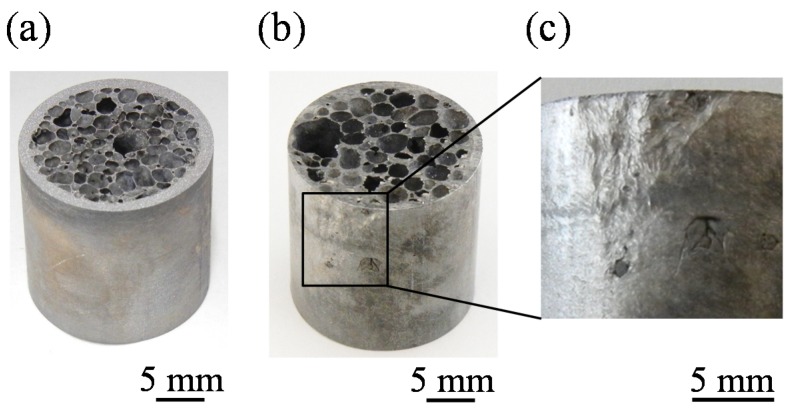
Fabricated ADC12-foam-filled A1050 tube with holding times of (**a**) *t*_H_ = 8.5 min and (**b**) *t*_H_ = 11 min; (**c**) Enlargement of part of (**b**).

Figure 4a shows the ADC12-foam-filled A1050 tube sample obtained with a holding time of *t*_H_ = 8.5 min. No cracklike defects were observed at the center part of the foam, where no precursor existed before foaming, or at the interface between the ADC12 foam and the A1050 tube. In addition, no deformation was observed for the tube, which retained its shape even after the foaming process. Figure 4b shows the ADC12-foam-filled A1050 tube sample obtained with *t*_H_ = 11 min. Although there were no defects in the foam or at the interface between the foam and the tube, it can be seen that the pores became large and that the infiltration of the foam into the tube occurred. Figure 4c shows an enlarged image of part of the surface of the tube in Figure 4b. Damage to the tube was observed, even at the surface, owing to the infiltration of the foam.

**Figure 5:**
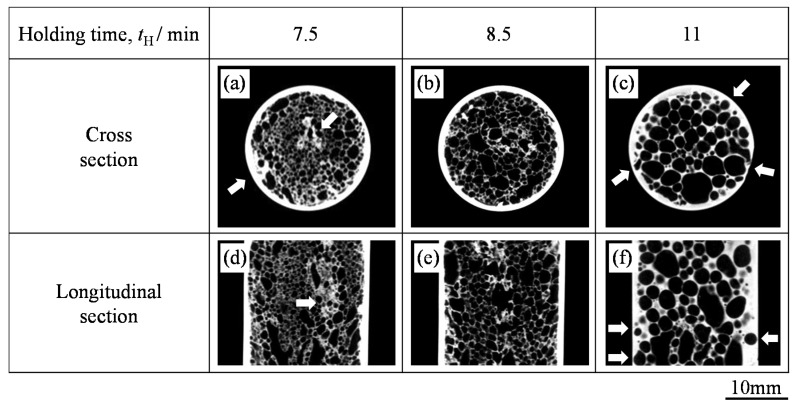
Cross-sectional and longitudinal-sectional X-ray CT images of fabricated ADC12-foam-filled A1050 tube samples with (**a**,**d**) *t*_H_ = 7.5 min, (**b**,**e**) *t*_H_ = 8.5 min, and (**c**,**f**) *t*_H_ = 11 min.

**Figure 6:**
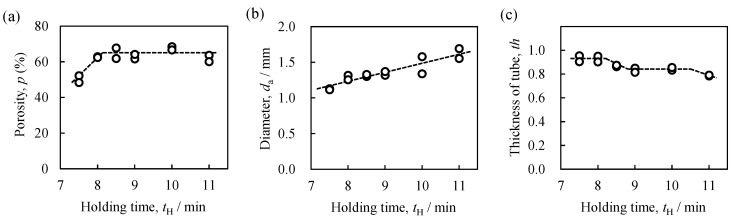
(**a**) Porosity *p*; (**b**) pore diameter *d*_a_; and (**c**) thickness of the A1050 tube *th* of fabricated ADC12-foam-filled A1050 tubes as function of holding time *t*_H_.

Figure 5 shows typical X-ray CT images of the center of the cross section and the longitudinal section of ADC12-foam-filled A1050 tube samples fabricated with various holding times. The outer round white circles in Figure 5a–c indicate the A1050 tube and the white parts inside the white circles indicate the cell walls of the ADC12 foam. Figure 6a–c respectively show the porosity *p* of the foam, the average equivalent diameter of pores *d*_a_ in the foam, and the average thickness of the tube *th*, in ADC12-foam-filled A1050 tube samples plotted against *t*_H_, which were evaluated from cross-sectional X-ray CT images such as those in Figure 5a–c. For *t*_H_ = 7.5 min, as shown in Figure 6a,b, the porosity was low and the pores were small. Furthermore, some dense regions existed near the center of the foam and at the boundary between the tube and the foam as shown by the arrows in Figure 5a,d. These results indicate that the precursor was not sufficiently softened for the pores to grow and expand the precursor. For *t*_H_ = 8.5 min, as shown in Figure 5b,e, it can be seen that the foam completely filled the interior of the tube and the pores were distributed almost homogeneously. No dense regions showing insufficient foaming were observed. As shown in Figure 6a,b, the porosity was higher and the pores were larger than those for *t*_H_ = 7.5 min. No gap was observed between the foam and the inner wall of the tube, indicating that the precursor remained in contact with the tube during the foaming process. When the holding time was further increased, the porosity became almost constant while the pore diameter increased because some pores coalesced into large pores. This tendency was also consistent with the photographs and X-ray CT images of the ADC12-foam-filled A1050 tubes shown in Figure 4 and Figure 5. In addition, the foam began to infiltrate into the tube, as shown by the arrows in Figure 5c,f. It can be seen that the thickness of the tube gradually decreased with increasing holding time, especially at *t*_H_ = 11 min, as shown in Figure 6c. Consequently, it was shown that a holding time of approximately *t*_H_ = 8.5 min was suitable for obtaining sound ADC12-foam-filled A1050 tubes with sufficient foaming and minimum reduction in the thickness of the tube.

**Figure 7:**
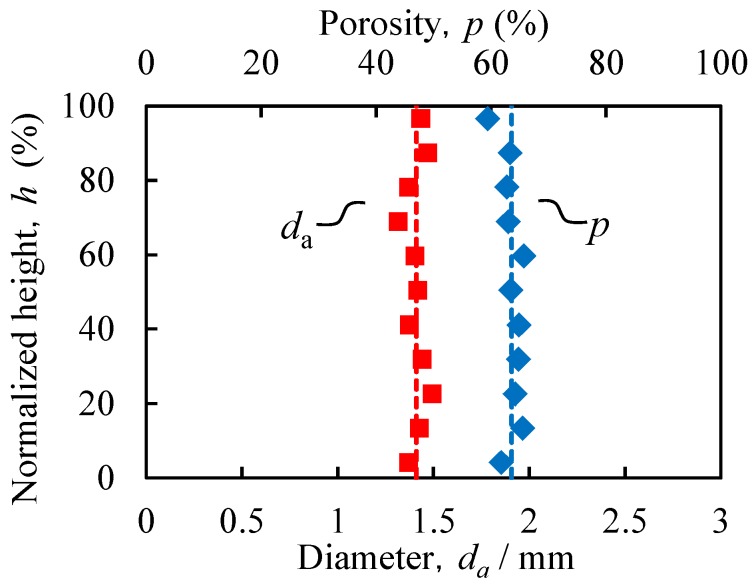
Relationship between specimen height and average diameter *d*_a_ of pores and porosity *p*.

Figure 7 shows the relationship between the location in the ADC12-foam-filled A1050 tube (*t*_H_ = 8.5 min), expressed as the height *h* from the bottom of the specimen normalized by the specimen height, and the average diameter *d*_a_ of the pores and the porosity *p*. It can be seen that the obtained ADC12-foam-filled A1050 tube has almost uniform pore structures in the entire region.

### 2.3. Distribution of Elemental Si

**Figure 8:**
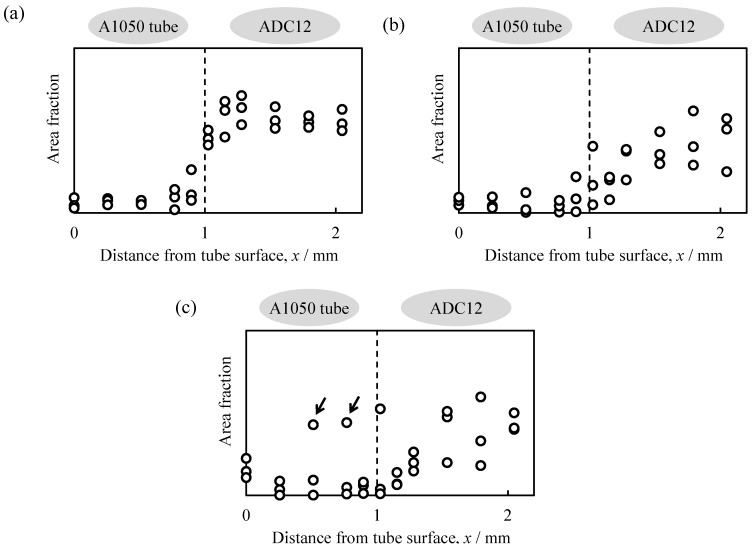
Distribution of the area fraction of elemental Si as a function of distance from the surface of the A1050 tube *x* for (**a**) ADC12-precursor-bonded A1050 tube; (**b**) ADC12-foam-filled A1050 tube with *t*_H_ = 8.5 min; and (**c**) ADC12-foam-filled A1050 tube with *t*_H_ = 11 min.

Figure 8a–c show the distributions of the area fraction of elemental Si around the interface between the A1050 tube and the ADC12 part as a function of the distance from the outer surface of the tube *x* in the ADC12-precursor-bonded A1050 tube shown in Figure 3b and in the ADC12-foam-filled A1050 tubes with *t*_H_ = 8.5 min and *t*_H_ = 11 min, respectively. Note that the initial thickness of each tube was 1 mm; therefore *x* = 1 mm, shown by dotted lines in Figure 8, indicates the initial inner surface of the tube. As shown in Figure 8a, the Si content in the ADC12-precursor-bonded A1050 tube increased abruptly from the A1050 tube to the ADC12 precursor at *x* = 1 mm, and the interface between the tube and the precursor can be clearly observed. The Si content in the ADC12-foam-filled A1050 tube with *t*_H_ = 8.5 min increased gradually from the A1050 tube to the ADC12 foam. However, the Si content on the tube side was similar to that in the ADC12-precursor-bonded A1050 tube; therefore, it is considered that the infiltration of the foam and the diffusion of the elemental Si did not proceed further.

As can be seen in Figure 8c, there were some areas with a high elemental Si content in the A1050 tube with *t*_H_ = 11 min as shown by arrows. It is considered that the infiltration of the foam and the diffusion of the elemental Si proceeded further as *t*_H_ increased. In a previous study, two types of ADC12-foam-filled steel tubes were fabricated. In one type, the precursor was bonded to the steel tube by friction welding, and in the other type, the precursor was not bonded to the steel tube and was only placed in the steel tube and foamed. Although it appeared that the same ADC12-foam-filled steel tube was obtained, metal bonding between the ADC12 foam and the steel tube was only realized in the sample subjected to friction welding. In contrast, the Al foam was easily removed from the steel tube for the ADC12-foam-filled steel tube sample not subjected to friction welding [20]. This was considered to be because friction heat was generated and the strong plastic flow of the ADC12 precursor occurred continually for a few minutes, which resulted in the formation of a new surface at the interface and the precursor attached firmly to the tube. Considering the results shown in Figure 6c and Figure 8 and also the previous study, it was concluded that metal bonding between the A1050 tube and the ADC12 foam was achieved with the minimum infiltration of the foam into the tube when *t*_H_ was 8.5 min. The bonding of the ADC12 precursor to the A1050 tube was necessary to fabricate the Al-foam-filled tube, which realized sufficient bonding between the foam and the tube. Duarte *et al.* reported that *in situ* foam-filled aluminum alloy tubes, which were prepared by heating the Al precursor inside Al tubes, had narrow gaps between the Al foam and the inner Al tube surface [28]. In addition, the attachment of the ADC12 precursor to the A1050 tube by friction welding is important to prevent the deformation and reduction of the thickness of the tube during the foaming process. Because the ADC12-foam-filled A1050 tube not subjected to friction welding, the fracture of the oxide layer of the surface at the interface is necessary during the foaming process to realize sufficient bonding, in which deformation and the infiltration of the foam into the tube may occur.

## 3. Experimental Section

### 3.1. Fabrication Process of ADC12-Foam-Filled A1050 Tube

**Figure 9:**
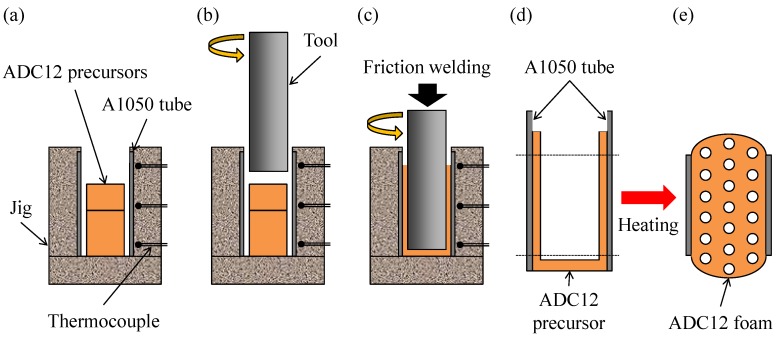
Schematic illustration of fabrication process of ADC12-foam-filled A1050 tube by friction welding. (**a**) Precursors were set in the tube; (**b**) A rotating tool was inserted into the tube; (**c**) The tool was inserted and friction welding was conducted; (**d**) ADC12-precursor-bonded A1050 tube was obtained; (**e**) ADC12-foam-filled A1050 tube was obtained.

Figure 9 shows a schematic illustration of the fabrication process of an ADC12-foam-filled A1050 tube. First, as shown in Figure 9a, an A1050 tube with 1 mm wall thickness, 20 mm outer diameter, and 40 mm height was set in a fixing jig. Then, two ADC12 precursors with dimensions of 9.5 mm × 16 mm × 24 mm and 9.5 mm × 16 mm × 11 mm, into which blowing agent powder had been previously mixed, were set in the tube. Next, as shown in Figure 9b, a rotating tool made of SUS304 steel of 13 mm diameter was inserted into the tube from above at a feed rate of 5 mm/min and a rotation speed of 800 rpm. As shown in Figure 9c, the tool was inserted until its tip was 3 mm above the bottom of the tube, *i.e.*, the rotating tool was moved downward by a further 32 mm after first coming into contact with the surface of the top ADC12 precursor. Friction heat was generated between the precursors and the rotating tool during friction welding, and the plastic flow of the softened precursors resulted in the filling of the gap between the tool and the A1050 tube. The temperature during friction welding was measured using thermocouples placed at upper, middle, and lower positions in the fixing jig located 2 mm from the surface of the tube. Finally, the obtained ADC12-precursor-bonded A1050 tube, whose upper and lower parts were welded to obtain a height of 30 mm as shown in Figure 9d, was placed in a preheated electric furnace held at 948 K, resulting in the fabrication of the ADC12-foam-filled A1050 tube as shown in Figure 9e. The holding time *t*_H_ in the furnace (foaming time) was varied from *t*_H_ = 7.5 min to *t*_H_ = 11 min. Two samples were foamed for each holding time. The upper and lower parts of the foamed samples were welded by an electrodischarge machining to obtain ADC12-foam-filled A1050 tube samples with dimensions of *φ* = 20 mm × 20 mm.

**Figure 10:**
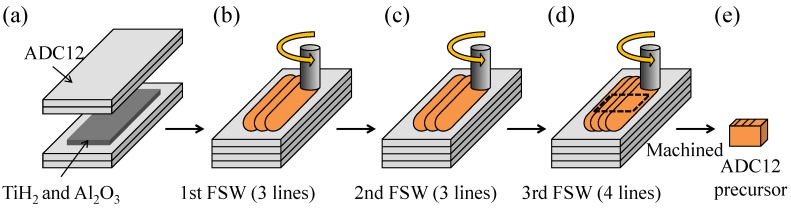
Schematic illustration of fabrication process of ADC12 foam precursor using FSW route. (**a**) The powders were placed along the path of the FSW tool; (**b**) FSW was conducted. 1st FSW; (**c**) 2nd FSW; (**d**) 3rd FSW; (**e**) Precursors were obtained.

Figure 10 shows a schematic illustration of the fabrication process of the ADC12 precursor by the FSW route [29,30]. As the starting materials, ADC12 high-pressure die-cast plates of 3 mm thickness were used. First, as shown in Figure 10a, four ADC12 plates were stacked with TiH_2_ powder (<45 μm, 1 mass %) as the blowing agent and Al_2_O_3_ powder (~1 μm, 5 mass %) as the stabilization agent distributed between the second and third plates. Next, as shown in Figure 10b–d, FSW was performed on the laminated plates to mix the powders in the ADC12 plates and to join the plates. FSW was carried out using an FSW machine (Hitachi Setsubi Engineering Co., Ltd., Hitachi, Japan). The FSW tool was cylindrical and had a screw probe. The diameter of the tool shoulder was 19.5 mm, the diameter of the tool probe was tapered from 8 mm to 4 mm, and its length was 9.3 mm. The tool rotation speed was 1000 rpm and the welding speed was 50 mm/min. A tilt angle of 3 deg was used. The multipass FSW technique, as shown in Figure 10b–d, was applied three times to the FSW region to be stirred to obtain a large amount of ADC12 precursor and to thoroughly mix the powders in the Al plates. Three passes (*i.e.*, three lines) were conducted for the first and second FSWs, and four passes (*i.e.*, four lines) were conducted for the third FSW. The ADC12 precursors of 9.5 mm × 16 mm × 24 mm and 9.5 mm × 16 mm × 11 mm used in the fabrication of the ADC12-foam-filled A1050 tubes were cut from the region subjected to FSW.

### 3.2. Observation of Deformation Behavior of Precursor and Evaluation of Pore Structures

To observe the deformation behavior of the ADC12 precursor during the friction welding and to evaluate the porosity *p* of the ADC12 foam part, the average diameter of the pores *d*_a_ of the ADC12 foam, and the thickness *th* of the A1050 tube of the ADC12-foam-filled A1050 tube, X-ray CT observations were performed on samples using an SMX-225CT microfocus X-ray CT system (Shimadzu Corporation, Kyoto, Japan). The X-ray source was tungsten. A cone-type CT system was employed to obtain a set of two-dimensional cross-sectional X-ray CT images of an entire specimen perpendicular to the A1050 tube with a slice pitch equal to the length of one pixel in the X-ray CT image by a single rotation of the specimen. The resolution of each X-ray CT image was 512 × 512. The pixel length was approximately 90 μm when examining the deformation behavior of the ADC12 precursor and approximately 70 μm when examining the ADC12-foam-filled A1050 tube. The X-ray tube voltage and current were 80 kV and 30 μA, respectively. For the evaluation of *p* and *d*_a_, the two-dimensional cross-sectional X-ray CT images were analyzed using WinROOF image processing software (Mitani Corporation, Fukui, Japan). An appropriate threshold was set to distinguish the cell walls and the pores in the two-dimensional cross-sectional X-ray CT images, and binarized X-ray CT images were obtained for the evaluation. Pores with diameter of less than 0.7 mm were excluded from the evaluation owing to the resolution of the X-ray CT images [31]. For the evaluation of *th*, the two-dimensional cross-sectional X-ray CT images were analyzed using ImageJ image processing software. *th* was taken as the average for ten images extracted at regular intervals from the obtained two-dimensional cross-sectional X-ray CT images of the entire specimen and was evaluated at four points in each image at intervals of 90 deg. Values of *th* exceeding 1 mm, which were obtained in the case that the cell walls of the ADC12 foam adjoined the A1050 tube, were considered to be 1 mm.

### 3.3. Observation of Microstructures

Elemental Si, which largely exists in ADC12 Al alloy but not in A1050 Al, was detected at the interface of the ADC12 part and the A1050 tube using an EPMA-1620 electron probe microanalyzer (Shimadzu Corporation, Kyoto, Japan). Samples of an ADC12-precursor-bonded A1050 tube and ADC12-foam-filled A1050 tubes with *t*_H_ = 8.5 min and *t*_H_ = 11 min were cut from the center part of a longitudinal section parallel to the A1050 tube, and the cut surface was observed. The observed surface was ground using SiC paper (of up to #2400) and then polished with alumina (1 μm) before the observation. EPMA mapping analyses were conducted over selected 128 × 128 μm^2^ areas along three lines from the surface of the A1050 tube to the ADC12 part. The area fraction of elemental Si in the EPMA mapping images was then obtained.

## 4. Conclusions

In this study, an ADC12-foam-filled A1050 tube was successfully fabricated by friction welding. First, the ADC12 precursor was firmly bonded throughout the inner wall of the A1050 tube without a gap between the precursor and the tube by friction welding. No deformation of the tube or foaming of the precursor was observed during the friction welding. Next, by heat treatment of an ADC12-precursor-bonded A1050 tube, an ADC12-foam-filled A1050 tube was obtained. A holding time during the foaming process of approximately *t*_H_ = 8.5 min with a holding temperature of 948 K was suitable for obtaining a sound ADC12-foam-filled A1050 tube with sufficient foaming, almost uniform pore structures over the entire specimen, and no deformation and minimum reduction in the thickness of the tube.

## References

[1] Gibson L.J. (2000). Mechanical behavior of metallic foams. Annu. Rev. Mater. Sci..

[2] Banhart J. (2001). Manufacture, characterisation and application of cellular metals and metal foams. Prog. Mater. Sci..

[3] Seeliger H.W. (2002). Manufacture of aluminum foam sandwich (AFS) components. Adv. Eng. Mater..

[4] Banhart J., Seeliger H.W. (2008). Aluminium foam sandwich panels: Manufacture, metallurgy and applications. Adv. Eng. Mater..

[5] Chung H.J., Rhee K.Y., Han B.S., Ryu Y.M. (2008). Plasma treatment using nitrogen gas to improve bonding strength of adhesively bonded aluminum foam/aluminum composite. J. Alloys Compd..

[6] Banhart J., Seeliger H.W. (2012). Recent trends in aluminum foam sandwich technology. Adv. Eng. Mater..

[7] Zu G., Song B., Zhong Z., Li X., Mu Y., Yao G. (2012). Static three-point bending behavior of aluminum foam sandwich. J. Alloys Compd..

[8] Hanssen A.G., Hopperstad O.S., Langseth M. (2000). Bending of square aluminium extrusions with aluminium foam filler. Acta Mech..

[9] Santosa S., Banhart J., Wierzbicki T. (2001). Experimental and numerical analyses of bending of foam-filled sections. Acta Mech..

[10] Toksoy A.K., Tanoglu M., Guden M., Hall I.W. (2004). Effect of adhesive on the strengthening of aluminum foam-filled circular tubes. J. Mater. Sci..

[11] Nishi S., Makii K., Aruga Y., Hamada T., Naito J., Miyoshi T. (2004). The manufacturing process and mechanical properties of porous aluminum. Kobe Steel Eng. Rep..

[12] Sun G.Y., Li G.Y., Hou S.J., Zhou S.W., Li W., Li Q. (2010). Crashworthiness design for functionally graded foam-filled thin-walled structures. Mater. Sci. Eng. A.

[13] Attia M.S., Meguid S.A., Nouraei H. (2012). Nonlinear finite element analysis of the crush behaviour of functionally graded foam-filled columns. Finite Elem. Anal. Des..

[14] Yin H.F., Wen G.L., Hou S.J., Qing Q.X. (2013). Multiobjective crashworthiness optimization of functionally lateral graded foam-filled tubes. Mater. Des..

[15] Yin H., Wen G., Wu X., Qing Q., Hou S. (2014). Crashworthiness design of functionally graded foam-filled multi-cell thin-walled structures. Thin-Walled Struct..

[16] Barnes T.A., Pashby I.R. (2000). Joining techniques for aluminium spaceframes used in automobiles Part II—Adhesive bonding and mechanical fasteners. J. Mater. Process. Technol..

[17] Hangai Y., Ishii N., Koyama S., Utsunomiya T., Kuwazuru O., Yoshikawa N. (2012). Fabrication and tensile tests of aluminum foam sandwich with dense steel face sheets by friction stir processing route. Mater. Trans..

[18] Hangai Y., Kamada H., Utsunomiya T., Kitahara S., Kuwazuru O., Yoshikawa N. (2014). Aluminum alloy foam core sandwich panels fabricated from die casting aluminum alloy by friction stir welding route. J. Mater. Process. Technol..

[19] Hangai Y., Saito M. (2013). Fabrication of an Al foam/dense steel composite by friction welding. Mater. Trans..

[20] Hangai Y., Saito M., Utsunomiya T., Kitahara S., Kuwazuru O., Yoshikawa N. (2014). Fabrication of aluminum foam-filled thin-wall steel tube by friction welding and its compression properties. Materials.

[21] Maalekian M. (2007). Friction welding—Critical assessment of literature. Sci. Technol. Weld. Join..

[22] Uday M.B., Fauzi M.N.A., Zuhailawati H., Ismail A.B. (2010). Advances in friction welding process: A review. Sci. Technol. Weld. Join..

[23] Shinoda T. (2011). Coating process using friction technology. Therm. Spray. Tech..

[24] Shinoda T. (2011). Applications of Friction Technology on Cast Materials. J. Jpn. Foundry Eng. Soc..

[25] Abu-Farha F. (2012). A preliminary study on the feasibility of friction stir back extrusion. Scr. Mater..

[26] Dinaharan I., Sathiskumar R., Vijay S.J., Murugan N. (2014). Microstructural characterization of pure copper tubes produced by a novel method friction stir back extrusion. Procedia Mater. Sci..

[27] Khorrami M.S., Movahedi M. (2015). Microstructure evolutions and mechanical properties of tubular aluminum produced by friction stir back extrusion. Mater. Des..

[28] Duarte I., Vesenjak M., Krstulović-Opara L., Anžel I., Ferreira J.M.F. (2015). Manufacturing and bending behaviour of *in situ* foam-filled aluminium alloy tubes. Mater. Des..

[29] Hangai Y., Kamada H., Utsunomiya T., Kitahara S., Kuwazuru O., Yoshikawa N. (2013). Compression properties of three-layered functionally graded ADC12 aluminum foam fabricated by friction stir welding. Mater. Trans..

[30] Hangai Y., Kamada H., Utsunomiya T., Kitahara S., Kuwazuru O., Yoshikawa N. (2014). Tensile properties and fracture behavior of aluminum alloy foam fabricated from die castings without using blowing agent by friction stir processing route. Materials.

[31] Hangai Y., Maruhashi S., Kitahara S., Kuwazuru O., Yoshikawa N. (2009). Nondestructive quantitative evaluation of porosity volume distribution in aluminum alloy die castings by fractal analysis. Metall. Mater. Trans. A.

